# Signature of a Pre-Human Population Decline in the Critically Endangered Reunion Island Endemic Forest Bird *Coracina newtoni*

**DOI:** 10.1371/journal.pone.0043524

**Published:** 2012-08-20

**Authors:** Jordi Salmona, Marc Salamolard, Damien Fouillot, Thomas Ghestemme, Jerry Larose, Jean-François Centon, Vitor Sousa, Deborah A. Dawson, Christophe Thebaud, Lounès Chikhi

**Affiliations:** 1 Société d’Etudes Ornithologiques de La Réunion (SEOR), St André, Ile de La Réunion, France; 2 Parc National de La Réunion, Saint Denis, Ile de La Réunion, France; 3 Instituto Gulbenkian de Ciênca, Oeiras, Portugal; 4 Department of Animal and Plant Sciences, University of Sheffield, Sheffield, United Kingdom; 5 Laboratoire Evolution and Diversité Biologique, Université Paul Sabatier, Toulouse, France; 6 Université de Toulouse; Toulouse, France; Leuven University, Belgium

## Abstract

The exceptional biodiversity of Reunion Island is threatened by anthropogenic landscape changes that took place during the 350 years of human colonization. During this period the human population size increased dramatically from 250 to 800,000. The arrival of humans together with the development of agriculture, invasive species such as rats and cats, and deforestation has lead to the extinction of more than half of the original vertebrate species of the island. For the remaining species, significant work is being carried out to identify threats and conservation status, but little genetic work has been carried on some of the most endangered species. In the last decade theoretical studies have shown the ability of neutral genetic markers to infer the demographic history of endangered species and identify and date past population size changes (expansions or bottlenecks). In this study we provide the first genetic data on the critically endangered species the Reunion cuckoo-shrike *Coracina newtoni*. The Reunion cuckoo-shrike is a rare endemic forest bird surviving in a restricted 12-km^2^ area of forested uplands and mountains. The total known population consists of less than one hundred individuals out of which 45 were genotyped using seventeen polymorphic microsatellite loci. We found a limited level of genetic variability and weak population structure, probably due to the limited geographic distribution. Using Bayesian methods, we identified a strong decline in population size during the Holocene, most likely caused by an ancient climatic or volcanic event around 5000 years ago. This result was surprising as it appeared in apparent contradiction with the accepted theory of recent population collapse due to deforestation and predator introduction. These results suggest that new methods allowing for more complex demographic models are necessary to reconstruct the demographic history of populations.

## Introduction

Using genetic data in combination with ecological data to inform conservation efforts has been standard practice for many years. Neutral genetic markers have proven very useful in describing genetic diversity both within and among populations, and for inferring their demographic history (e.g. [1]–[4]). It is also increasingly recognized that the separation of ancient and recent demographic events is crucial for the efficient management of endangered species [5]. For instance, a low genetic diversity could be the result of either a long-term small effective population size (*N_e_*) or a recent population collapse. In the latter case, it would be urgent to take management measures, whereas in the former case, management measures should focus on alternative conservation efforts other than ‘restoring’ genetic diversity. However, it is difficult to quantify the relative importance of ancient *versus* recent events since similar genetic patterns can be found as a result of very distinct demographic histories [6]–[8]. Nevertheless, in the last two decades, the use of different types of markers, such as microsatellites, combined with new population genetics analysis methods have shown that it is possible to detect genetic signatures of major demographic events, such as population collapses, admixtures and expansions, that have occurred in different time scales (e.g. [9]–[13]).

**Figure 1:**
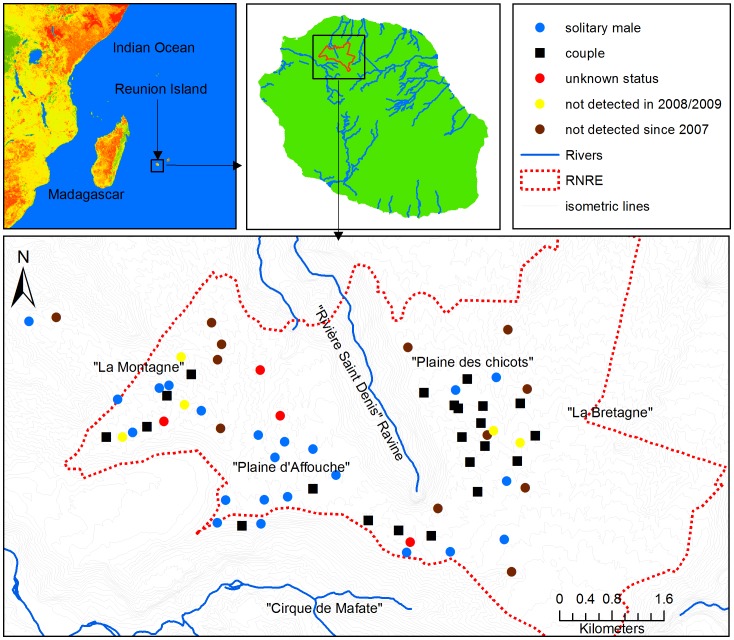
Geographical distribution of *C.newtoni*. This figure represents (a) the Reunion Island, (b) the “Roche Ecrite” National Reserve (c) *C.newtoni*’s known population in 2008–2009 and the status of the each known territory.

The Reunion Island (21°06′S; 55°32′E, 2512 km^2^, Figure 1a) is the youngest volcanic island of the Mascarene archipelago (around 2 million years old according to [14]). The Piton des Neiges is the former volcano that led to the formation of the island and it has experienced three explosive episodes around 230,000 years ago [15]. These episodes are thought to be responsible for the extinction of various species of birds having lost their ability to fly such as Rails (*Aphanapterix* and *Erythromachus*; [16]). More recent eruptions have also taken place during the Holocene [17], [18]. The island was discovered by Pedro de Mascarenhas in 1553 and the first inhabitants settled in 1663. The human population size then increased from 250 people in 1676 to around 800,000 today. The population increase suffered a period of stagnation between 1868 and 1950 due to recurrent malaria epidemics, a disease that was later eradicated by Dichlorodiphényltrichloroéthane (DDT) spraying between 1949 and 1962 [19]. The arrival of humans in the Reunion Island 350 years ago has generated major environmental changes and species extinctions. Lagabrielle *et al.* [20] estimated that 73% of the original vegetation cover was replaced by farmland (36%), urban areas (12%), and the 3500 introduced plant species (25%) of which 62 are highly invasive [21]. It is also estimated that 30 of the 45 species (67%) of vertebrates present on the island disappeared [16], [22] and that 42 vertebrate species were introduced, especially in highly modified habitats, amongst which rats (*Rattus norvegicus, R. rattus*) between 1672 and 1675 [22] and the domestic cat (*Felis catus*), introduced in 1703 [23], which are known to have contributed to many bird extinctions on islands [24], [25].

The Reunion Cuckoo shrike, *Coracina newtoni* Pollen 1866, locally called “Tuit tuit”, is the only bird of the Campephagidae group in Reunion Island. *C.newtoni* differentiated from *C. typica* of Mauritius island 1.25 million years ago [26]. With a current population estimated between 70 and 100 individuals, and composed of 48 territories with twenty-two of them occupied by couples and twenty six by solitary males [27], *C.newtoni* is the most highly endangered forest bird of Reunion island (status: Critically Endangered; [28]). Predation by cats and rats is known to be the main threat for adult survival and reproductive success [29]. Since 2003 conservation management is conducted with intensive rat control around the breeding nests following an action Plan for the conservation of the species [30]. The whole population is currently confined to a small area of 12 square kilometers (km^2^) in the north of the island, ranging from 1300 to 1800 meters in altitude. The current population habitat is mainly composed of mountain wet mixed evergreen forest and mountain *Acacia* forest [29], [31]. This area has been protected since 1999 by the creation of a Natural Reserve (RN “Roche Ecrite”; Figure 1). It is characterized by a rough topology and bisected from north to south by the “Rivière St Denis” ravine, which is considered as a natural geographical partial barrier that could affect the dispersal or gene flow pattern of *C.newtoni* population.

This study supplements the demographic and ecological knowledge already acquired on *C.newtoni* using different molecular tools, and use the case of *C.newtoni* to discuss the power and limitations of population genetic inference. We argue that genetic data while powerful should be interpreted with care when simple models are used and that species such as *C.newtoni* which are highly endangered provide important tests due to the fact that their recent history is reasonably well-known, despite many uncertainties on its past distribution.

## Results

### Genetic Diversity

As Table 1 shows, the allelic richness per locus (A_r_) was low (mean  = 3.82) and varied between 1.00 and 6.46 across loci, with 1 to 9 alleles per locus (Na) with an average of 4. Averaged expected heterozygosity (*H_e_*) across the sixteen polymorphic loci was found to be low with a value of 0.5 (Table 2) considering that these loci had been selected because they were polymorphic. Positive F_IS_ values were observed; however, none of the values were significantly different from zero for the population (P) data set suggesting that the sampled population is not deviating from Hardy-Weinberg equilibrium.

**Table 1:** Characteristics of the 17 microsatellite loci used to genotype cuckoo-shrike’s population.

Loci	Accession number	Alelle size range	Na	Ar	He	Ho	F_IS_
Ase18	AJ276375	188–190	2	2.00	0.5035	0.406	0.196
Asu15-ZEST	AY172993	118–120	2	2.00	0.4861	0.500	−0.029
CK.1B6G	AF026333	138–140	2	2.00	0.3791	0.413	−0.091
CK.5A5F	AF026338	123–125	2	2.00	0.4587	0.565	−0.235
CmeH2	AY330729	140–172	9	6.46	0.8065	0.783	0.030
MSLP4-ZEST	AB031376	145–151	4	3.80	0.6969	0.571	0.182
Pca3	AJ279805	163–179	3	1.69	0.0869	0.089	−0.023
PmaTGAn42	AY260540	276–292	5	3.98	0.7040	0.609	0.137
Ppi008	FM865709	328–348	7	5.25	0.7824	0.739	0.056
Ppi012	FM865713	253–261	5	3.17	0.5172	0.500	0.034
Ppi016	FM865717	210–214	3	2.07	0.2152	0.196	0.092
Ppi018	FM865719	127–143	7	5.16	0.7054	0.674	0.045
SAP47-ZEST	AY823673	391–391	1	1.00	–	–	–
TG02-088	DV579347	258–264	2	1.99	0.3562	0.370	−0.038
TG04-004	DV946288	170–172	2	2.00	0.4517	0.457	−0.011
Tgu07	DV948303	103–113	6	3.68	0.6531	0.761	−0.167
TGZ-037	DV945670	146–162	3	2.27	0.1936	0.207	−0.070

Microsatellite characteristics calculated using the 46 individuals.

Observed allele size range, number of alleles per locus across samples (N_a_), mean allelic richness per sample (A_r_), unbiased expected Heterozygosity (H_e_), observed Heterozygosity (H_o_) and F_is_ values for all loci and samples.

**Table 2:** Genetic diversity of *Coracina newtoni* population.

	P	K1	K2	K3
N	46	27	7	12
Na	3.82	3.12	2.82	3.12
Na#	4.00	3.25	2.94	3.75
Ar	2.97	2.81	2.68	2.87
Ar#	3.06	2.92	2.79	3.35
He	0.470	0.450	0.462	0.444
*SD* He	0.232	0.254	0.254	0.306
He#	0.500	0.478	0.491	0.500
*SD* He	0.217	0.233	0.230	0.278
Ho	0.461	0.458	0.479	0.444
Ho#	0.490	0.487	0.509	0.473
FIS	0.020	−0.018	−0.038	−0.001

Samples size per data set (N), average number of alleles per locus across samples (Na), mean allelic richness per sample (Ar), unbiased expected Heterozygosity (He), observed Heterozygosity (Ho), FIS and FST values for all samples (“P”) and the three identified clusters (“K1” to “K3”). *P<0.05, **P<0.01, ***P<0.001, sd standard deviation. #locus *SAP47-ZEST* not considered.

### Population Structure

#### Isolation by distance

Mantel test shows a significant correlation between Euclidean geographical distances and genetic inter-individuals distances (Pearson correlation coefficient R = 0.208, p value <0.001) suggesting a pattern of isolation-by-distance.

### Hidden Population Structure

The clustering method implemented in STRUCTURE 2.1 did not identify any hidden population substructure (Supporting Information SI) whichever model was used (with or without admixture, and with correlated or uncorrelated allele frequencies), suggesting that no strong population structure is detectable in *C.newtoni*’s population using this method.

**Figure 2:**
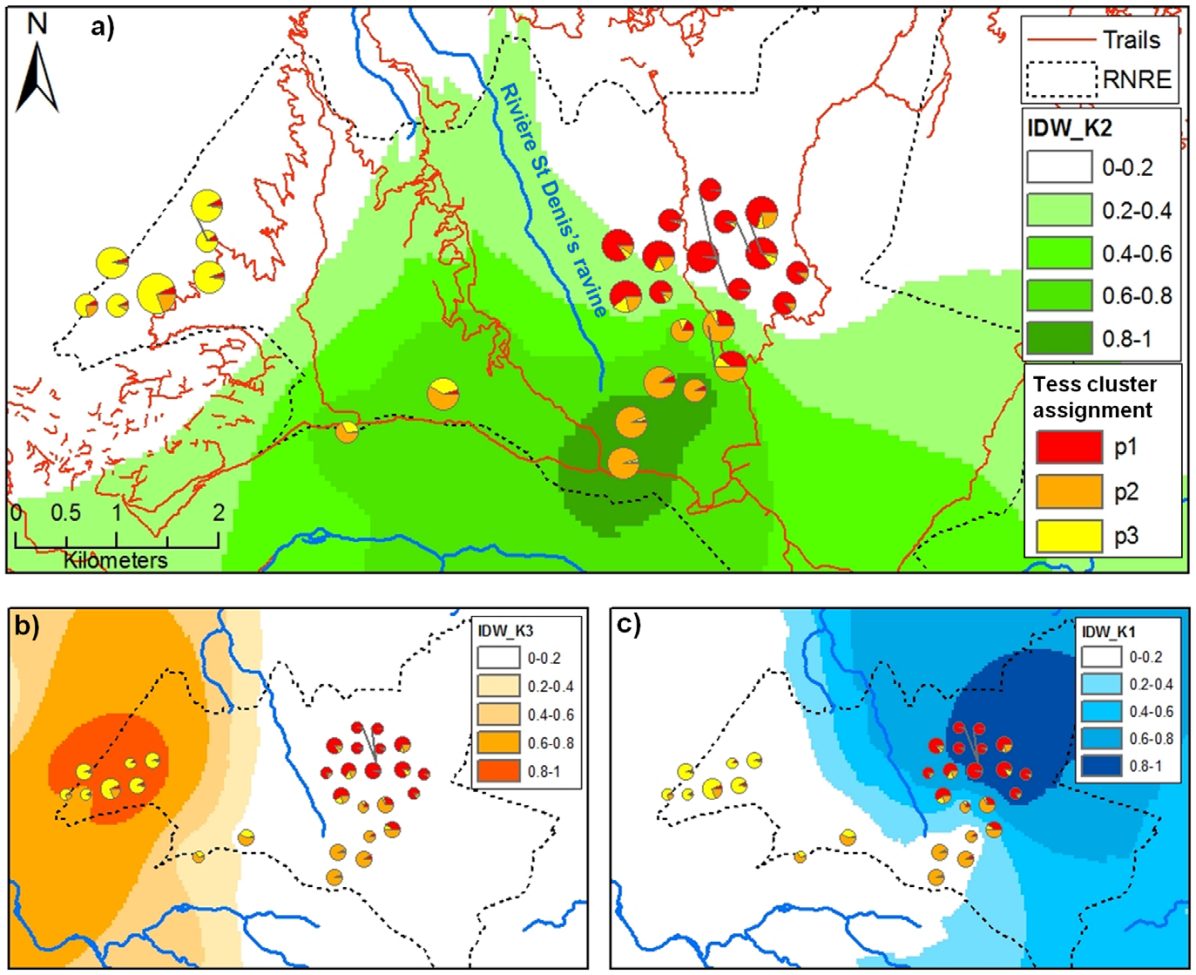
Membership of genetic clusters estimates using TESS. The size of the pie charts is proportional to the number of sampled individuals (between one and three individuals). The colors indicate the average membership coefficients of each individual to each of the three clusters uncovered by Tess. The gradients illustrate the interpolated (IDW) membership coefficients of the cluster K3 (a), K2 (b) and K1 (c) (see text for details).

Analysis performed with Francois *et al.* [32] method (implemented in the TESS software) shows lowest DIC values for K = 3 (Figure 2, Supporting Information SI). This result suggests that some substructure exists, grouping West, South-center, and North-East individuals in 3 clusters. In order to confirm the TESS K = 3 clustering result we performed a new STRUCTURE analysis using the TESS assignments as prior. The ΔK analysis of the results gave a most probable K value of 3 and assignment probabilities were very similar to the TESS analysis with higher probabilities for the members of each sub-groups (Supporting Information SI). In addition, this analysis shows the best K likelihood value of all the performed STRUCTURE analyses with and without prior information (Supporting Information SI) confirming two main results: (i) that STRUCTURE is strongly affected by the prior information, (ii) that it was unable to detect what appears to be either weak spatial substructure or just isolation by distance. Interestingly, the pairwise F_ST_ values calculated between the clusters were all significant and ranged from 0.044 for K1–K2 to 0.079 for K2–K3 (Supporting Information SI) again confirming the detection of a substructure using TESS. Nevertheless, these results are likely to be affected by the small size of our sample. Indeed, as shown in Figure 1 and 2 our study lacks samples in “Plaine d’Affouche” and “La Bretagne”. An increased sampling of the population would be required to accurately refine the patterns of genetic structure in *C.newtoni*’s population.

### Demographic History

#### Detection of expansions and/or declines

As population structure is known to confound demographic events inference [2], [33], [34], [8], [7], we tested all demographic methods using both the whole population (“P”), and the three detected cluster (“K1” to “K3”). The results of the Wilcoxon test for *H_e_* excess showed that there was a significant signal for a population decrease, for all the data set tested, under the IAM. Under the TPM, the signal was significant for the K1 and K2 clusters. However, no significant departure was detected under the SMM. No departure from mutation-drift equilibrium was detected using the M-Ratio analysis. (Supporting Information SI).

**Figure 3:**
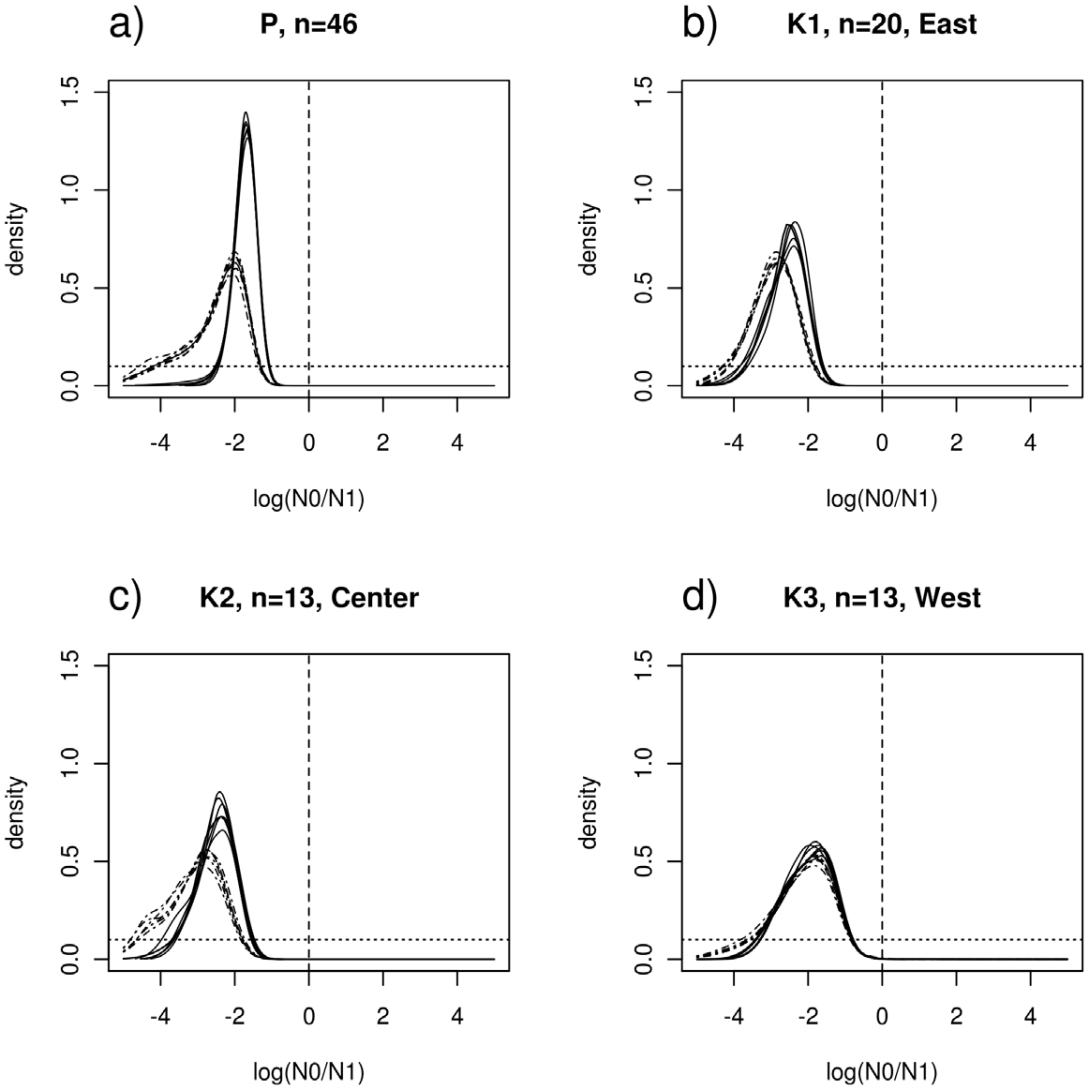
Demographic collapse detected using MSVAR 0.4. Posterior distributions of the effective population size change, log(N_0_/N_1_). Solid lines correspond to the exponential population size change model. Dashed lines correspond to the linear population size change model. Log(N_0_/N_1_) represents the ratio of present (N_0_) to past (N_1_) population size. The dashed vertical line corresponds to the absence of population size change (Log(N_0_/N_1_) = 0). The prior distribution is shown for comparison (flat dotted line).

Using the full-likelihood Beaumont (1999) method we found a clear signal of population collapse whatever the dataset used (P, K1, K2 or K3), and the demographic model (exponential or linear; Figure 3), with no support for growing or even stable demographic history. The posterior of log_10_(*N_0_/N_1_*) indicated a decrease in effective population size of about two orders of magnitude (Figure 3), with a current population size approximately 200 times smaller than the population size before the start of the decrease (mean(*N_0_*/*N_1_*) = 0.005; Supporting Information SI). The results were similar whether we used or excluded the monomorphic locus (SAP47-ZEST) and whether we used only half of the loci (data not shown). The weight of evidence of the hypothesis that a population decline occurred vs. a population increase, were assessed using approximate “Bayes factors” (BF), i.e., the ratio of the posterior densities of the two alternative hypotheses, over the ratio of the prior densities of the same alternative hypotheses. BF calculated for the population decrease hypothesis (H1) show very high values (>68) under the exponential model and higher than 5 (between 5 and 18) under the linear model. Given that BF>3 and 10 are usually considered to indicate “positive” and “significant” evidence, respectively [35], this provides a very strong support for a population decrease under both the exponential and linear models but shows also that some sensitivity to model assumptions also exists (Supporting Information SI).

**Figure 4:**
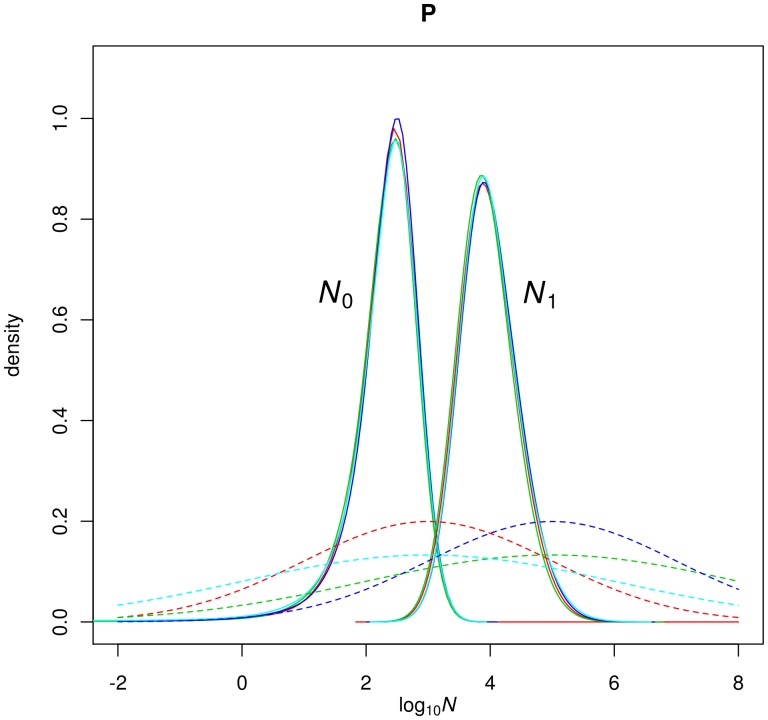
Posterior distributions for the past (N_1_) and present (N_0_) effective population sizes. N_1_ and N_0_ are represented on a log_10_ scale. The solid lines correspond to the posterior distribution obtained by pooling independent MCMC run. The different priors used are shown for comparison for N_0_ and T, (dashed lines) and for N_1_ (doted- lines).

Using the Storz & Beaumont (2002) method, we found similar results. The posterior distributions of log(*N*_0_) and log(*N*_1_) have very limited overlap for the whole population “P” (Fig. 4) and the K1 to K3 clusters (Supporting Information SI), with respective medians of approximately 2.4 (*N*_0_∼250) and 3.9 (*N*_1_∼8500; Supporting Information S1). Note that the ratio of these median values is not necessarily the same as the median of the ratio, and should not be compared to the value of *r* obtained above. Altogether these values indicate a strong collapse of *C.newtoni* populations. All the posteriors are very different from the priors and converged approximately to the same distributions whichever the priors used, which strongly suggests that the data contain a signal for a population decrease. Moreover, BFs calculated for the population decrease hypothesis shows a very high value of 54.5 (Supporting Information SI). The log(*N*_0_) posterior distributions point to a reduced present effective population size, with most values concentrated between approximately 1.26 and 3.27 on log scale (95% Highest posterior density – HPD), corresponding to *N_0_* values between 18 and 1862, respectively, and with natural scale median values of respectively 256. The log(*N_1_*) posterior distributions show higher values with most HDP values concentrated between approximately 3.06 to 4.93 corresponding to 1,148 to 85,000, respectively and with natural scale median values of 8,520 (Supporting Information SI).

### Timing of Population Size Change

**Figure 5:**
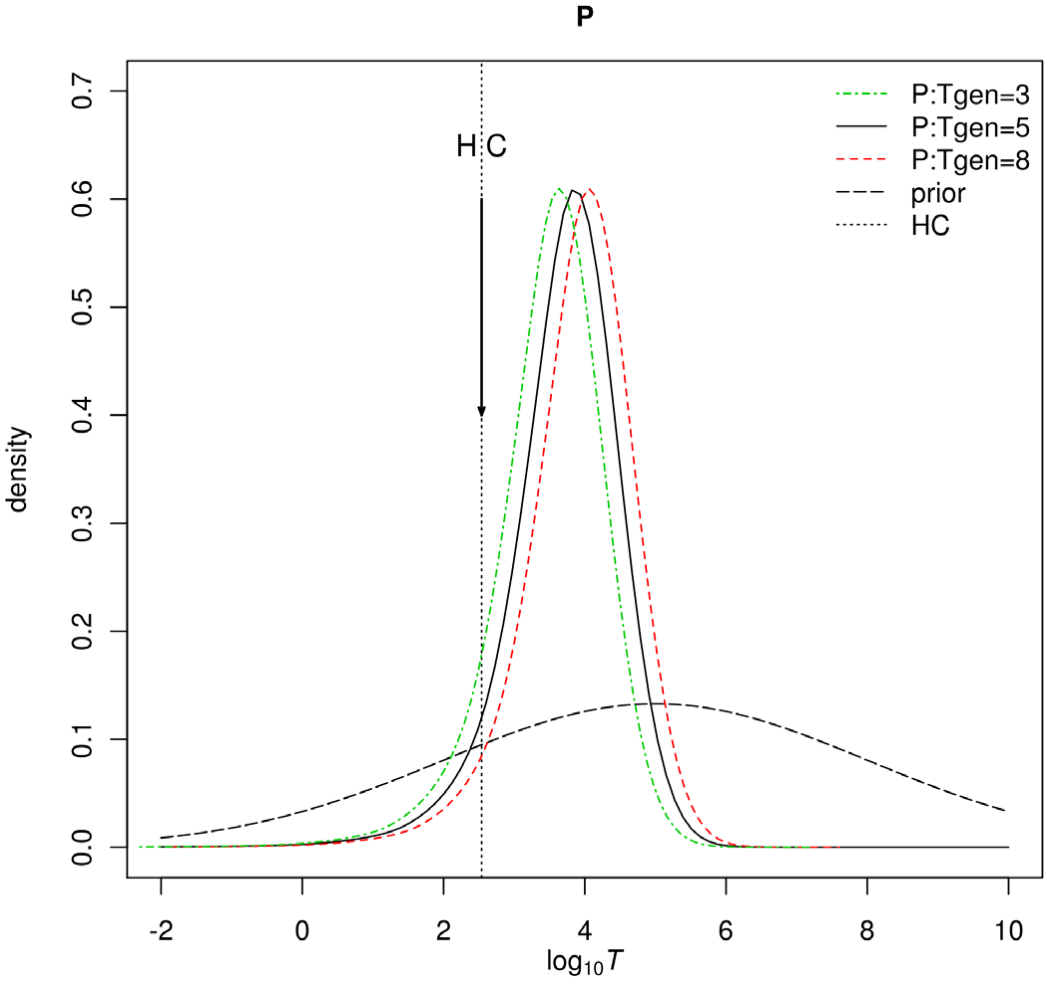
Time since the population collapse. The posterior distribution for the time since the population collapse started is represented on a logarithmic scale. These distributions have a median around 6,000 years BP. Most of their mass is concentrated in recent years with a sharp decrease as time goes back. The prior is shown as a dashed line, its median being 100,000 y ago (see text). The arrow corresponds to the human colonization (HC) of the island in 1664.

**Figure 6:**
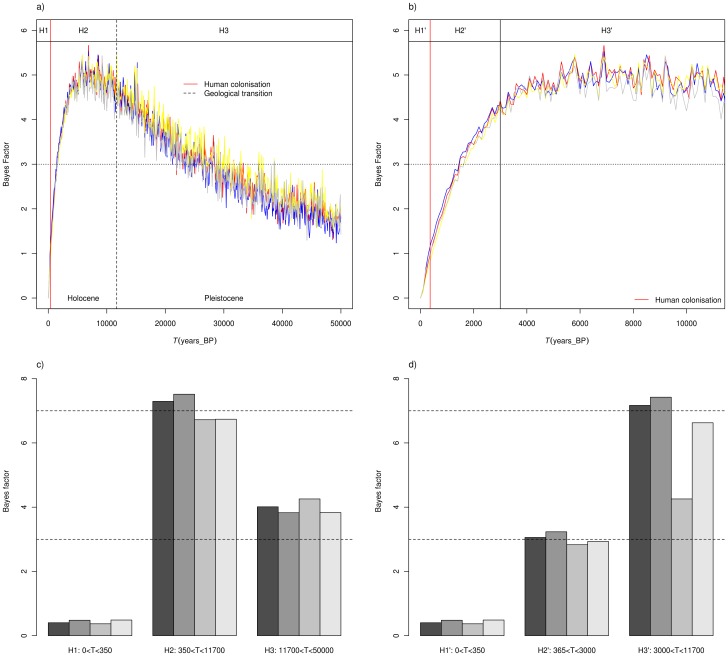
Most likely period since the population size started to decrease. Natural logarithm of the Bayes factors have been computed to determine during which period *C.newtoni* population most likely started to decline. BF values of parameter *T* (time since the beginning of the demographic event) for the whole population (P) were computed for each 100 year time steps and are plotted for the last 50,000 (a), and 11,700 years (b). This represents the weighted evidence in favor of each phase against the cumulative evidence of all other phases taken together. Horizontal dashed-dotted lines represent the threshold values above which BF values can be considered as positive evidence [35]. Vertical black red line corresponds to the year 1663 in which the Island was colonized by the first humans. H1 to H3 and H1’ to H3’ represent the six time period hypothesis tested in c and d. In panel c) and d) bar plot represent BF values of parameter *T* of each time period hypothesis (H1 to H3 and H1’ to H3’) against the cumulative evidence of the two other phases taken together, for each of the four independent runs. Horizontal dashed-dotted lines represent the threshold values above which BF values can be considered as positive evidence [35].

Using MSVAR1.3, the posteriors of log(*T*), the time since population started to decrease, shows a mode around 3.8 (*t* = 6,300 years; Figure5) for a generation time of 5 years and a distribution skewed to the left, with 50% HPD between 2,200 and 17,000 years and 90%HPD between 300 and 71,000 ybp, whichever prior distribution was used. The BF analysis of *T* (Figure 6a) shows the highest values during the Holocene, before human colonization of the island. Analysis of the weighted evidence in favor of each phase against the cumulative evidence of all other phases taken together (Figure 6c) confirms that Holocene is the period with the highest support (BF>7). A refined BF analysis of *T* posteriors (Figure 6b, and Figure 6d) within the Holocene suggests that the most probable period for the population collapse is between 3,000 and 12,000 years before present (BP).

## Discussion

### Evidence for a Pre-human Bottleneck in *Coracina newtoni*

The demographic history of endangered species and populations is often complex, and can result from recent, anthropogenic related pressures, and from more ancient expansions and contractions caused by humans or natural factors. The latter include climatic and environmental fluctuations such as droughts, fires, floods, volcanic eruptions and even cyclones in Indian Ocean islands. All of these factors (natural and anthropogenic) can generate both local and global contraction in habitat and population size. Tropical islands such as La Réunion are particularly interesting, as the history of human colonization is well known and very recent, making it easier to separate ancient (pre-human) from recent human factors. The results obtained with the full-likelihood methods suggest that *C.newtoni* had been subjected to a major population contraction of perhaps two orders of magnitude. This result was less clear with methods based on summary statistics, such as Bottleneck and M-P val. This is not surprising as these methods use less information than MSVAR which uses the full allelic distribution. This has been known for a long time but has been confirmed recently by a simulation study which showed that it does not detect old events [36]. For *C. newtoni*, the Bottleneck analyses showed no strong or consistent signal for a departure from mutation drift equilibrium under the different mutation models (Supporting Information SI). Since the mutation process of our microsatellites is to certain extent unknown, a significant heterozygosity excess for all mutation models would have been considered as a strong evidence of a recent population decline, as was found in orangutans [11]. We found that assuming the I.A.M. for microsatellites lead to higher rates of bottleneck detection than when assuming the S.M.M. or T.P.M. and similar discrepancies between mutation models have been found in other studies [1], [36], [37].

The absence bottleneck signal detection using M-P val (Supporting Information SI) was not surprising. This method has been shown to efficiently detect bottlenecks [38], to have low type II error rates, even for small values of *θ* [39] and to be relatively robust compared to Bottleneck, even for old population declines [36], but it is still based on only one summary statistics.

Note that all the methods used here are based on the information present in the allelic distribution (either using summaries or the full information) and are thus unable to detect events older than 4Ne generations (on average). This means that the effective size of *C. newtoni* cannot have been too small for very long periods. For instance, if Ne = 50 and if the generation time is five years, then bottlenecks older than 1,000 years would not be detected. The results of MSVAR thus clearly point to the fact that *C. newtoni* cannot have maintained very small populations for long periods. For instance a bottleneck around 5000 years requires that Ne>200 for a significant part of the last 5,000 years. Otherwise the population would have reached equilibrium and no population size change would be detected. Since there are less than 100 individuals today and probably an Ne<50, this is a strong indication that *C. newtoni* had a larger distribution on the island when humans arrived and that there was probably a recent decline.

Another crucial and related point to mention is that all methods used here were tested or developed under the assumption that there was only one demographic event. As we discuss below, bibliographic and ecological data suggest that the population probably suffered several contractions. To our knowledge no study has yet been performed to assess the behavior of these methods in the case of multiple demographic events, but we feel that this would be necessary to make the interpretation of genetic bottlenecks more reliable.

Given the current population size of *C.newtoni*, below 100 individuals, and its distribution in a high altitude environment considered as sub-optimal [29], detecting a bottleneck based on genetic data is far from surprising. Indeed, historical data of the last decades, together with early naturalists’ reports and descriptions and recent studies suggest that (i) the distribution of *C.newtoni* was wider than today, (ii) the species was more abundant, (iii) cuckoo-shrikes were easy to catch when humans arrived some 350 years ago, and (iv) their population has then been impacted by introduced cats and rats. First, as shown in Supporting Information SI, the population’s lowest altitude limit seems to have shifted from 800 meters in 1865 to 1,300 meters in 2008 [27], [29], [40]. Second, while the species was first described as “abundant” in 1665, it was defined as Critically Endangered in 2008 [28], [40] due to a population size of less than 100 individuals. But note that the population trend during this period is confusing (Supporting Information SI). Third while Pollen managed to capture 14 individuals with nets in 1865, it is extremely difficult to capture individuals today [27], [40], [41] suggesting that the reaction of the species to potential threats and predators has developed during this period. Fourth, cats and rats have been clearly identified as major predators of *C.newtoni* [27], [29], and the population seems to slowly increase since the protection against rats and cats started [27]. Indeed the number of occupied territories, breeding pairs, and of successful reproduction events increased during this period [27]. When introduced at the end of the 17^th^ century, rats and cats probably first colonized areas inhabited by humans, and only later spread to wild habitats. There is however no data on cats and rats densities across the Reunion Island but one could easily imagine that the reduction in the distribution area of *C.newtoni* mentioned above could have result from the colonization and the increase of the population of these two predators in midlands area.

We should also mention that early naturalists (Bourreau-Deslandes, 1676; Le Gentil, 1717; de Cordemoy, 1860 cited in [42], [43]) reported the presence of a “grive”, (a French term for thrush, and a general vernacular name for species belonging to six bird genera of the *Turdidae* family: *Turdus*, *Catharus*, *Hylocichla*, *Brachyptera*, *Zoothera*, and *Nesocichla*). “Grives” generally exhibit morphological similarities with *C.newtoni*, but no species of those genera were later formally described in La Réunion Island. Interestingly this “grive” was described as common, very abundant, and was found in lowland areas and easy to catch with a wood stick [42]. Assuming that this “grive” could be *C.newtoni*, the earlier distribution area of *C.newtoni* would thus be much wider than it is now (see [42] for bibliographic review of this hypothesis). Nevertheless the hypothesis that the early described “Grive” was in fact *C.newtoni* is still controversial, and more archeological data are required to determine if a thrush-like bird or *C.newtoni* were present in the lowlands.

This historical information suggests that *C.newtoni* was subjected to a human driven population decline in the last centuries. However, together with the detection of genetic bottlenecks found in the present study, it does not produce a straightforward picture. Indeed, one of the most interesting results of our genetic analysis came from the dating of the population crash with the MSVAR 1.3 method. While it is difficult to identify the precise date or period during which *C.newtoni* population declined (most likely between 3,000 and 10,000 years ago, Figure 4 and 5), our results clearly identify a pre-human Holocene event which does not fit with a recent human-driven contraction.

Historically, the period identified for the bottleneck signal is not unrealistic, since several dry periods appear to have taken place during the Holocene, the warmest and driest of which took place *ca*. 4500 years ago and probably lasted several centuries [44]. This period has also been identified as a period of severe drought in Madagascar [45] with a significant increase in fires and an expansion of grasslands and savannas (see also [46]). In Réunion Island, increasing temperature together with dry periods could have constrained or restricted the population to the wetter upland forests, reducing *C.newtoni*’s populations and geographical distribution to high altitudes in a situation, perhaps, not unlike the current one. In addition, volcanic records suggest an explosive activity of the Piton de la Fournaise around 5,000 to 3,000 years ago [17], [18]. Taken together, this suggests that *C.newtoni* may have been subjected to various population contractions during the Holocene, but it remains unclear whether we can identify the event (or the combination of events) that may have caused the bottleneck signal, or whether volcanic or climatic events are the more likely culprits.

Altogether our results suggest that the *C.newtoni* population has been maintained at a relatively small size during the last millennia on perhaps several occasions. As noted above the population must have had a Ne>200, otherwise no signal could have been detected with MSVAR. How long the periods with small Ne were is difficult to determine, but it is interesting to note that several studies have identified cases with long term survival of small populations. These studies are still limited but they suggest that long time persistence is not inconsistent with low levels of genetic diversity or small population size in vertebrates species [12], [47]. As discussed in Reed et al. 2010 [47] for the Madagascar fish eagle (*Haliaeetus vociferoides*) data from Johnson et al. 2009 [12], the probability for a population to persist at such a small size (120 pairs) and for a such long period (>200 generations), is negligible. Our results suggest that a vertebrate species can go through small population sizes and persist on an island for more than 1,000 generations. Reed et al. [47] suggested that the long term survival of a small population is more likely to occur in benign environments. Even if Reunion Island cannot be considered as a benign environment due to the presence of volcanic eruptions, and cyclones, one could easily hypothesize that this improbable persistence had occurred thanks to the absence of terrestrial predators. This hypothesis could also explain the dramatic impact of the recent introduction of terrestrial predators on *C.newtoni*.

It is noteworthy that population genetic inference may be affected both by the sampling strategy and by the caveats and assumptions of the methods used here. The sampling issue is often neglected but will certainly require more attention in the future. In our case, one limitation is that our sample size was necessarily small due to the current size of this critically endangered bird and to the difficulty to catch adults. As a consequence, our sampling included chicks sampled in a relatively small number of nests (n = 29). The effect of this biased sampling is to our knowledge unknown, in the same way that sampling social groups, or related individuals, as is done for many species is often ignored. However, we believe that this should not have had a major effect in our case because we repeated the analyses for different sampling strategies and always found the same results. However, we do think that the use of related individuals or social groups as source of potential bias in population genetics inference is understudied. Beyond the sampling effect, departures from the demographic model may also cause improper inference with few exceptions (e.g. [7], [48]–[50]) most inferential methods have been developed to account for only one major demographic event (e.g. a single bottleneck), and have been tested only under very limited conditions due to their high computational costs. Hence it remains unclear how estimates are affected by deviations from model assumptions, such as multiple past bottlenecks. Chikhi *et al.* [8] have recently shown that the method implemented in MSVAR can be sensitive to population structure, because most methods developed to identify bottlenecks assume that the samples obtained in the field can be approximated by a Wright-Fisher model, i.e. that there is no population structure. While this may be a reasonable approximation in some cases, Chikhi *et al.* [8] have shown that even under high gene flow conditions some data sets exhibit clear bottleneck signatures, even though the simulated populations were stationary. However they have also shown that this effect can be accounted for by analyzing individuals from several populations. In *C.newtoni* we identified a bottleneck also when we analyzed all individuals together, which suggest that the bottleneck signal is not due to population structure alone.

In the present study, we used MSVAR 0.4 to determine whether *C.newtoni* had been subjected to a population size change and MSVAR1.3 to estimate past and present population sizes and to date this decline. But since these methods only allow for one population size change event and have not been tested with data sets from populations submitted to multiple population size changes, it is difficult to interpret our results in a straightforward manner. For instance, do the methods identify the most important bottleneck in the recent past or do they identify a “virtual” bottleneck summarizing the combined effect of several bottlenecks? Moreover, the fact that population structure generates spurious bottlenecks [7], [8]suggests that the effect detected may also be partly due to population structure or isolation by distance [33]. It is of course not possible to simulate data sets under all possible scenarios to test the robustness of new inferential methods. However, the recent development of Approximate Bayesian Computation (ABC) model choice procedures may provide potential solutions to this problem. A recent study has shown that a very large number of independent loci (100) would be necessary to identify the true model with high certainty [7].

In a recent simulation study testing MSVAR 1.3, Girod *et al*. [36] have shown that the method identifies more easily ancient compared to very recent population collapses. Moreover, it was shown that for a population decline to be detected the pre-bottleneck population must be at mutation-drift equilibrium. Given that several periods of major environmental changes probably affected *C.newtoni* during the Holocene, the population may not have had time to reach equilibrium when humans arrived. Indeed, the estimated effective population size before the collapse (*N_1_*) with a mean/median around 9,000 individuals (90% HPD values between 1,300 and 24,000; Supporting Information SI) suggests that the population was distributed across the whole island. After a population contraction a few thousand years ago followed by a re-expansion across the island, a new equilibrium should only be reached after thousands of generations. Thus, when humans arrived, they would have caused the collapse of a species that was still not at equilibrium.

Altogether, we believe that our data identify an old population bottleneck that is independent of the recent arrival of humans in Réunion. This does not mean that the genetic make-up of *C.newtoni* was not impacted by the recent anthropogenic habitat loss, but that the genetic data are the result of several demographic events and that ancient bottleneck are likely to have left the strongest mark. This also suggests that theoretical work is needed to better understand how space (*i.e.* structure, isolation by distance, spatial expansions/contractions) and time (recent *versus* ancient events) influence the genetic make-up of species.

### Towards the Conservation of *C.newtoni*

Despite its low population size, our results suggested that *C.newtoni* exhibit a limited but still detectable isolation by distance pattern (even though STRUCTURE could not detect it without prior information). Our study suggests that there is a possible barrier effect of the “Rivière St Denis’s ravine, that separates *C.newtoni*’s distribution in two main but small regions (la Plaine d’Affouche and la Montagne in the West, and La Plaine des Chicots and La Bretagne in the East; Figure 1 and 2) that communicate by a small 500 meter wide corridor (l’Entonnoir). This IBD is in agreement with field observations that show limited dispersion with young *C.newtoni* as they build nests at an average distance of 360 m from their parent’s nests, and always less than 1.2 km [27].

*C. newtoni* is currently characterized by a very small distribution, and population size, compared to other *Coracina* species (see [43] for review), and its habitat above 1300 m is considered as sub-optimal [29]. Indeed, using the distribution of forest types and habitats estimated by Strasberg *et al.* [51] before the arrival of humans in La Reunion, and known population densities of *C.newtoni* and of other *Coracina* species, we extrapolated the ancient probable population size for different densities, habitat and repartition hypotheses (Supporting Information SI). The results are in agreement with the estimated pre-bottleneck population size (with a mean/median *N_1_*∼9,000 individuals) (Supporting Information SI), and they suggest that the species was probably common across all upland wet forests or all wet forest of the island with respective population sizes between 6,000 and 18,000 and between 9,000 and 26,000 individuals (H2–H3 in Supporting Information SI).

Beyond the ancient demographic event signal detected in the genetic data, the population appears to have suffered a recent contraction. Indeed, the population was very close to extinction less than ten years ago and only survived thanks to the associations that implemented predator control and continue monitoring of the last population of *C. newtoni*.

### Conclusion

In this study we analyzed the population genetic patterns of one of the most endangered passerine bird of the Reunion Island. We found limited levels of genetic diversity, and population structure. We also found a strong signal for an ancient population bottleneck, prior to the arrival of humans in the Réunion Island. While we cannot identify the main cause for this signal, it suggests that *C.newtoni* was not at equilibrium when humans arrived on the island and that this is likely to be true of other endemic species. We discussed several potential issues that may have influenced these results and call for the development of ABC methods tailored to address these issues. This includes the analysis of the properties of models with several bottlenecks, and the effect of several bottlenecks on methods that only assume one demographic event. Another issue that also needs to be addressed in the future is the bias caused by the selection of the markers (i.e. ascertainment bias [52]). Clearly more work is needed to better understand how genetic data behave in time and space.

## Materials and Methods

### Sampling

Blood sampling was conducted in the “Reserve Naturelle de la Roche Ecrite” (Reunion Island, fig. 1). As the capture of adult individuals is very difficult [27], [41], and, in order to limit disturbance on reproduction, most of the blood samples (40 out of 46) were collected from chicks a few days before their first flight. In the 29 sampled nests, multiple (2–5) chicks were sampled, in the same year (12 nests, 26 chicks) and in subsequent years (3 nests, 9 chicks). In addition, blood samples were collected from two adults and four dead chicks following predation. Given the small size of the population and the low number of nestlings identified each year, sampling took place in each breeding season from 2003 to 2008 (except 2005; Supporting Information SI). Thus, to our knowledge, at least four of the sampled chicks could have been produced by two adults previously sampled. This study was approved by the “Comité consultatif de la Réserve de la Roche Ecrite” (arrêté 1096 du 14 mai 2006). Birds handling and sampling were permitted under the convention protocol of the 28^th^ of April 2003. DNA extraction details are described in Salmona *et al.* [53].

### Microsatellites

All sampled individuals were typed for 17 microsatellites (Table 1) selected from a set of 110 loci developed in passerine species and tested for cross amplification in *C.newtoni* [53]. The methods used to amplify and type the microsatellite loci in *C.newtoni* are also described in [53].

### Genetic Diversity

Genetic diversity was measured as the number of alleles (n_A_), allelic richness (A_r_), observed heterozygosity (H_o_), and unbiased expected heterozygosity (H_e_) estimated according to Nei [54]. Departures from linkage equilibrium, estimated with the correlation coefficient of Weir [55], were assessed with 10,000 permutations.

Deviation from Hardy-Weinberg equilibrium and genetic differentiation between populations were estimated using Wright’s F-statistics F_IS_ and F_ST_, respectively, according to the method of Weir and Cockerham [55]. Deviations from the respective null hypotheses were estimated with 10,000 permutations. These analyses were performed using GENETIX 4.05.2 [56] with the exception of the allelic richness, which was calculated using HP-RARE [57].

### Population Structure

The existence of population genetic structure was examined using both correlation analysis and clustering approaches. First we searched for the existence of patterns of relatedness and of isolation by distance in the population, and finally we used two Bayesian model-based clustering approaches developed by Pritchard *et al.* [58] and François *et al.* [32], respectively. These methods aim at detecting the structure of a genetic sample with or without prior information on the geographical origin of individuals. Hence, they do not fully depend on the units defined by our sampling strategy and try to recover any hidden partition in the data.

### Isolation by Distance

The *a_r_* inter-individual genetic distance [59] was computed using the program SPAGeDI [60] and a Mantel test [61] was performed using GenAlex [62] to determine if the patterns of differentiation follow isolation-by-distance [63]. Isolation by distance (IBD), was also investigated using Moran’s I spatial autocorrelation coefficient [64].

### STRUCTURE Analysis

The Pritchard *et al.* [58] and Falush *et al.* [65] method, implemented in STRUCTURE 2.3, groups individuals into genetic clusters using a Markov chain Monte Carlo (MCMC) approach, regardless of their geographical origin. In order to estimate the number *K* of genetically differentiated populations, we ran the program for a range of *K* values between one and six, and analyzed the distribution of the ‘estimated likelihood of *K*’ for each clustering result, which is an *ad hoc* approximation of the likelihood of *K*. The maximum *K* value was chosen as the number of potential sampled fragment plus three, as suggested by Evanno et al. [66]. We ran the program under the admixture and no admixture models considering both independent and correlated allele frequencies. We also ran the analysis under the recently developed model implemented in the 2.3 version of the STRUCTURE software, which infers weak population structure with the assistance of sample group information [67]. For each *K* value, each model and each parameter, we performed 50 independent runs with a 2×10^4^ burn-in period followed by 10^5^ steps. The most probable *K* value was determined by applying the Evanno *et al.* [66] *ad hoc* summary statistic *ΔK*, which is based on the rate of change of the “estimate likelihood” between successive *K* values. Evanno *et al.* [66] have shown that their method detects the highest hierarchical structure level. We thus repeated the analysis for each of the clusters identified in the previous step until no substructure was observed (*K* = 1).

### TESS Analysis

The Francois *et al.* [32] method implemented in TESS also groups individuals into *K* homogeneous clusters (populations) using a HMRF (Hidden Markov Random Field) and takes into account prior geographical distribution of samples. We used TESS in addition to STRUCTURE because its algorithm is expected to be less influenced by Isolation-By-Distance [32]. The network neighborhood created by TESS on the basis of geographical data was modified to not allow connections through the “Rivière St Denis” ravine, potential geographic barrier. We conducted an analysis for values of *K* populations of 1 to 6, with 100 runs for each value of *K*. For each run, 10^5^ steps, preceded by 3×10^4^ *burn-in* period were performed, as suggested by François *et al.* [32]. The 20 best runs were chosen according to their DIC values and the individual’s assignment probabilities of the 20 runs were compiled using CLUMPP [68] and plotted using Distruct1.1 [69]. Each individual was then assigned to the cluster for which the posterior probability was the highest, provided that this value was superior to 0.6, as commonly used [70], [71]. Mean membership assignment probabilities of each locality were computed, and the inverse distance weighted (IDW) interpolation function implemented in ArcGis 9.2 was used to predict the overall pattern of cluster membership through the species range.

### Demographic History

The detection of changes in effective population size was investigated using two different but complementary approaches as in Goossens *et al.* [11]. The first uses summary statistics (expected heterozygosity, allelic range and number of alleles), to detect population size changes, while the other set of methods are full-likelihood Bayesian coalescent methods that permit to detect, quantify and date the changes in population effective size based on the allele frequencies.

In the first approach, we investigated (i) the method developed by Cornuet and Luikart [72] based on expected heterozygosity excess implemented in BOTTLENECK 1.2.02 program [73] and (ii) the M-ratio method developed by Garza and Williamson [38] implemented in M_P_Val program. Using Cornuet and Luikart [72] method to test for significant deviations from the null hypothesis (stationary population), 10,000 *H_e_* values were simulated and compared to the observed values, using the Wilcoxon Sign Rank Test, under three mutational models: infinite allele model (I.A.M.), stepwise mutation model (S.M.M.), and a two-phase model (T.P.M.), in which, 30% of mutations were allowed to occur in a multi-step manner. Finally, the M-ratio method was investigated assuming the stepwise mutation model (S.M.M.) in which 10% of mutations were allowed to occur in a multi-step manner with a mean step size of 3.5 as recommended by Williamson-Natesan [39]. The M-ratio value obtained with the observed data was then compared to critical Mc values calculated for *θ = *10 and *θ = *1 using 10,000 permutations of the allele distribution data.

The Beaumont (1999) method implemented in the MSVAR 0.4.2 program assumes that a stable population of size *N_1_* started to decrease (or increase) *ta* generations ago to the current population size, *N_0_*. The change in population size is assumed to be either linear or exponential, and mutations are assumed to occur under a SMM model, with rate *θ* = 2*N_0_*µ, where µ is the locus mutation rate. Using a Bayesian coalescent-based MCMC approach, the method estimates the posterior probability distributions of (1) the magnitude of population size change *r = N_0_*/*N_1_*, (2) the time since the population started changing size *t_f_ = t_a_*/*N_0_*, scaled by *N_0_*, and (3) the scaled mutation rate *θ* = 2*N_0_*µ.

The method uses the information present in the full allelic distribution allowing the quantification of the population increase or decrease. However, this cannot be dated since time is scaled by *N_0_*, which remains unknown. For each sampled population the analyses were performed both under the linear and exponential models and six independent runs were performed, using different parameter configurations, starting values and random seeds. In this method, wide uniform prior distributions were chosen (between –5 and 5 on a log_10_ scale) for log(*r*), log(*Θ*), and log(*t_f_*) (Supporting Information SI). Positive log(*r*) values, corresponding to a population expansion, were set as the MCMC starting point (Supporting Information SI). The total number of iterations was always larger than 5×10^9^ with a thinning interval of 5×10^4^ (Supporting Information SI).

We also used the method developed by Storz & Beaumont [10] implemented in the MSVAR 1.3 program to quantify the effective population sizes *N_0_* and *N_1_*, as well as the time *T* since the population change (in generations). In order to express time in years we considered three different generation time for *C.newtoni* (3, 5 and 8 years). These approximated values has been chosen based on (limited) field observations showing that (i) one year old females can reproduce but seems to fail before their third year, (ii) females seem to choose preferentially older males (iii) some old (10 and 12 years old) birds have recently been reproducing successfully, and (iv) older birds seem to have lower reproductive success. These data still need to be confirmed and refined by the ongoing population monitoring. In this model, prior distributions for *N_0_*, *N_1_*, *T*, and *Θ*, are assumed to be normal on a log_10_ scale, i.e. lognormal. Wide ‘uninformative’ priors and multiple runs with different starting points and different hyperprior parameters were used. At least 4 runs were performed for each sample with a total number of iterations always larger than 4×10^10^ steps (Supporting Information SI). Different sets of priors were used to test their influence on the posteriors, but in most of the runs we set prior means for *N_0_*, *N_1_*, *T* (on a log_10_ scale) with means 4.0, 4.0 and 5.0, respectively; varying the standard deviations between 1 and 5 (Supporting Information SI). For *Θ* we set a mean of −3.5 with standard deviation of 0.25, so that values for the mutation rate in the region 10^–4^ to 10^–3^ had reasonable support, as widely assumed in demographic analysis [10] (Supporting Information SI). For both the Beaumont [74] and Storz & Beaumont [10] methods, the first 10% of each independent analysis were discarded to avoid influence in parameter estimation by starting conditions (*burnin* period). The convergence of the three runs for each sample was checked both visually and using Geweke convergence diagnostic [75] and results were grouped to obtain precise estimates of the posterior distributions.

Since we were interested in separating anthropogenic from other factors in causing signals of population collapse we tried to estimate the relative probability of recent *versus* ancient events by determining whether the data favored events that were older or more recent than *T* = 350 years. In practice, the weights of evidence of the hypothesis that time is <350 years *vs.* >350 years, were assessed using approximate “Bayes factors” (BF), *i.e*., the ratio of the posterior probability of the two alternative hypothesis, over the ratio of the prior probability of the same two alternative hypothesis. BFs greater than 3 indicate positive evidence and greater than 10 are considered significant [35].

Finally, we also estimated, using ecological data, the range of ancient (pre-bottleneck) *C. newtoni* populations. We used the Reunion Island ancient and current vegetation classification data from Strasberg et al 2005 [51] and five habitat type hypotheses were considered (see Supporting Information SI). Two population density hypotheses were considered, in the first the ancient density is the same as the current density (one bird pair *per* 12 ha), in the second the population density is twice higher (i.e. assuming that there is a greater resource availability at lower altitudes). This would be in agreement with the smaller home range size of other *Coracina* species (i.e.: one bird pair per 3.5Ha for the sister species *C.typica* in Mauritius Island; [27], [29]). In the higher density hypothesis an unbiased sex ratio was also considered, hence increasing the population size by 2/3.

## Supporting Information

Supporting Information S1 (DOC)
