# Supplementary material for: Signature of a Pre-Human Population Decline in the Critically Endangered Reunion Island Endemic Forest Bird Coracina newtoni
Source: PLoS One. 2012 Aug 20;7(8):e43524. doi: 10.1371/journal.pone.0043524 (PMC3423348; doi:10.1371/journal.pone.0043524)
Supplement: Supporting Information S1 — (DOC) [file pone.0043524.s001.doc]

**SUPPLEMENTARY DATA**

**Table S1**: STRUCTURE clustering likelihood results.

| K value | 1 | | 2 | | | 3 | | | 4 | | | 5 | | | 6 | | |
| --- | --- | --- | --- | --- | --- | --- | --- | --- | --- | --- | --- | --- | --- | --- | --- | --- | --- |
| Parameter set | mean | SD | mean | SD | Δ(k) | mean | SD | Δ(k) | mean | SD | Δ(k) | mean | SD | Δ(k) | mean | SD | Δ(k) |
| Without prior | **-1321** | **0.5** | -1455 | 67.0 | 2.6 | -1542 | 155.2 | 1.6 | -1507 | 183.8 | 1.4 | -1478 | 177.7 | 1.5 | -1516 | 175.3 | 0.9 |
| 2k Ravine prior | -1321 | 0.7 | **-1292** | **2.9** | 41.7 | -1382 | 40.8 | 2.6 | -1373 | 42.5 | 1.8 | -1366 | 56.1 | 2.0 | -1351 | 51.9 | 1.2 |
| 2k sampling gap prior | -1321 | 0.4 | **-1289** | **1.9** | 64.3 | -1379 | 38.2 | 3.1 | -1370 | 44.7 | 2.2 | -1356 | 43.8 | 2.1 | -1330 | 34.8 | 1.3 |
| 3k sampling gap prior | -1321 | 0.6 | **-1290** | **1.6** | 49.6 | -1337 | 30.7 | 2.0 | -1364 | 43.2 | 2.7 | -1403 | 77.2 | 2.1 | -1369 | 65.6 | 1.4 |
| 3k admx samples | -1321 | 0.5 | **-1289** | **1.4** | 62.4 | -1356 | 37.9 | 2.4 | -1353 | 45.1 | 1.9 | -1351 | 47.8 | 1.9 | -1332 | 39.0 | 1.3 |
| 2k random prior | **-1321** | **0.7** | -1373 | 59.5 | 1.8 | -1358 | 48.7 | 1.8 | -1338 | 22.7 | 1.9 | -1333 | 14.5 | 1.5 | -1327 | 6.5 | 1.5 |
| 3k TESS assignment prior | -1321 | 0.4 | -1291 | 3.2 | 4.9 | **-1276** | **4.1** | **17.0** | -1330 | 49.4 | 1.8 | -1343 | 53.1 | 1.8 | -1330 | 40.7 | 1.3 |

Mean and standard deviation (SD) of Likelihood values (L(K)) obtained from STRUCTURE analysis under admixture model and correlated allele frequencies. Highlighted values correspond to the most probable K (number of genetic cluster) value determined by the Evanno *et al.* (2005) ad hoc summary statistic Δk. Parameter set correspond to the tested priors.

**Table S2: Genetic differentiation (Fst values) for each pair of identified clusters.**

| Cluster | K1 | | K2 | | K3 | |
| --- | --- | --- | --- | --- | --- | --- |
| K1 |  |  |  |  |  |  |
| K2 | 0.063 | ** |  |  |  |  |
| K3 | 0.079 | *** | 0.044 | *** |  |  |

* P<0.05, ** P<0.01, *** P<0.001

**Table S3**: Heterozygosity excess and allele distribution demographic.

|  | Wilcoxon | | |  | Mode shift | | | | M-Ratio |  |  |  |  |
| --- | --- | --- | --- | --- | --- | --- | --- | --- | --- | --- | --- | --- | --- |
| Mutation model | P | K1 | K2 | K3 | P | K1 | K2 | K3 |  | P | K1 | K2 | K3 |
| IAM | **0.01** | **0.01** | **0.00** | **0.05** | **Normal L-shaped distribution** | | | | M | 0.82 | 0.79 | 0.81 | 0.87 |
| TPM | 0.11 | **0.03** | **0.03** | 0.26 |  |  |  |  | Mc *Ө*=10 | 0.72 | 0.67 | 0.64 | 0.64 |
| SMM | 0.67 | 0.25 | 0.12 | 0.40 |  |  |  |  | Mc *Ө*=1 | 0.80 | 0.80 | 0.79 | 0.79 |

P-values for the heterozygosity excess given by the ‘Wilcoxon signed rank test’ obtained under three mutation models (I.A.M. infinite allele model, T.P.M. two-phase model, S.M.M. stepwise mutation model). P-values < 0.05 indicate population decrease (bottleneck). Values in bold indicates significant detection of a demographic contraction event. Results of the mode shift analysis of allele’s frequencies (a normal L-shape distribution indicates no signal of population decrease). M-Ratio calculated (M) can be considered as a signal of a demographic contraction event if lower than estimated M-ratio critical values (Mc).

**Table S4: Posterior *N0/N1* values of the bottleneck signal obtained with MSVAR 0.4**

| Parameter |  | Log10(*N0/N1*) | | | | | | | | | | |  | Log10(*Tf*) | | | | | | | | | | |
| --- | --- | --- | --- | --- | --- | --- | --- | --- | --- | --- | --- | --- | --- | --- | --- | --- | --- | --- | --- | --- | --- | --- | --- | --- |
| Sample |  | P | |  | K1 | |  | K2 | |  | K3 | |  | P | |  | K1 | |  | K2 | |  | K3 | |
| Model |  | exp | lin |  | exp | lin |  | exp | lin |  | exp | lin |  | exp | lin |  | exp | lin |  | exp | lin |  | exp | lin |
| Mean |  | -2.34 | -4.44 |  | -3.59 | -4.12 |  | -3.58 | -4.64 |  | -3.21 | -3.96 |  | 0.38 | 1.98 |  | 0.31 | 1.94 |  | 0.47 | 2.38 |  | -0.02 | 1.21 |
| Median |  | -1.24 | -1.51 |  | -1.80 | -2.02 |  | -1.72 | -1.98 |  | -0.99 | -1.01 |  | 0.37 | 1.73 |  | 0.31 | 1.85 |  | 0.46 | 2.20 |  | -0.04 | 0.97 |
| q(0.95) |  | -1.75 | -2.58 |  | -2.60 | -2.97 |  | -2.54 | -3.13 |  | -2.01 | -2.20 |  | -2.34 | -4.44 |  | -3.59 | -4.12 |  | -3.58 | -4.64 |  | -3.21 | -3.96 |
| q(0.05) |  | -1.71 | -2.35 |  | -2.53 | -2.92 |  | -2.48 | -3.02 |  | -1.96 | -2.08 |  | 0.79 | 3.97 |  | 0.53 | 3.17 |  | 0.79 | 4.08 |  | 0.40 | 3.69 |
| var |  | 0.13 | 0.78 |  | 0.30 | 0.41 |  | 0.32 | 0.64 |  | 0.52 | 0.86 |  | 0.06 | 0.93 |  | 0.02 | 0.42 |  | 0.03 | 0.75 |  | 0.14 | 1.07 |
| HPD 95 lower |  | -2.43 | -4.58 |  | -3.71 | -4.25 |  | -3.72 | -4.84 |  | -3.38 | -4.23 |  | -0.06 | 0.56 |  | 0.04 | 0.76 |  | 0.13 | 1.06 |  | -0.56 | -0.25 |
| HPD 95 upper |  | -1.10 | -1.30 |  | -1.60 | -1.76 |  | -1.53 | -1.86 |  | -0.80 | -0.69 |  | 0.86 | 4.11 |  | 0.57 | 3.30 |  | 0.84 | 4.25 |  | 0.51 | 3.95 |
| HPD 50 lower |  | -1.89 | -2.54 |  | -2.79 | -3.24 |  | -2.77 | -3.35 |  | -2.32 | -2.43 |  | 0.19 | 0.90 |  | 0.23 | 1.40 |  | 0.33 | 1.46 |  | -0.17 | 0.40 |
| HPD 50 upper |  | -1.47 | -1.63 |  | -2.09 | -2.40 |  | -2.05 | -2.30 |  | -1.37 | -1.37 |  | 0.50 | 1.95 |  | 0.40 | 2.12 |  | 0.57 | 2.49 |  | 0.11 | 1.25 |
| Mode |  | -1.93 | -2.03 |  | -2.87 | -3.53 |  | -2.53 | -3.11 |  | -2.06 | -2.31 |  | 0.11 | 2.00 |  | 0.14 | 2.03 |  | 0.25 | 1.77 |  | -0.21 | 1.19 |
| BF H1 |  | 1421 | 9.21 |  | 68.83 | 14.13 |  | 85.33 | 5.06 |  | 252 | 18.49 |  | - | - |  | - | - |  | - | - |  | - | - |
| BF H2 |  | 0.00 | 0.00 |  | 0.00 | 0.00 |  | 0.00 | 0.00 |  | 0.00 | 0.00 |  | - | - |  | - | - |  | - | - |  | - | - |

Values of quantile 0.05 (q.05), quantile 0.95 (q.95), mean, median, variance (var) and highest posterior density (HPD) of the posterior distribution of *N0/N1* (current effective population size / old effective population size before the demographic event) and of *Tf* =*T.N_0._*generations (Unscaled parameter composed by *T*, the Time at which the population started to decrease, *N_0_* the current effective population size, and the generation time of the species) obtain with the Beaumont 1999 method for analysis performed with exponential (exp) and linear (lin) population size change models. Bayes factor for population decline and increase hypothesis (respectively BFH1, and BFH2) are given as a basis of comparison to Chikhi et al (2010) and Girod et al (2011) tests of the model.

**Table S5: Posterior values and accuracy of the bottleneck signal obtained with MSVAR 1.3.**

|  | Sample | P |  |  |  | K1 |  |  |  | K2 |  |  |  | K3 |  |  |
| --- | --- | --- | --- | --- | --- | --- | --- | --- | --- | --- | --- | --- | --- | --- | --- | --- |
| Generation time | | 3 | 5 | 8 |  | 3 | 5 | 8 |  | 3 | 5 | 8 |  | 3 | 5 | 8 |
| *N*0 | Median | 256 |  |  |  | 18 |  |  |  | 68 |  |  |  | 171 |  |  |
|  | Mean | 208 |  |  |  | 12 |  |  |  | 43 |  |  |  | 85 |  |  |
|  | q.05 | 23.5 |  |  |  | 0.1 |  |  |  | 0.6 |  |  |  | 0.7 |  |  |
|  | q.95 | 1033 |  |  |  | 301 |  |  |  | 647 |  |  |  | 1140 |  |  |
| Log10(*N*0) | HPD 95 lower | 1.26 |  |  |  | -0.98 |  |  |  | -0.32 |  |  |  | -0.29 |  |  |
|  | HPD 95 upper | 3.27 |  |  |  | 2.80 |  |  |  | 3.11 |  |  |  | 3.37 |  |  |
|  | HPD 50 lower | 2.19 |  |  |  | 0.93 |  |  |  | 1.60 |  |  |  | 2.04 |  |  |
|  | HPD 50 upper | 2.75 |  |  |  | 2.25 |  |  |  | 2.67 |  |  |  | 2.94 |  |  |
|  | Mode | 2.41 |  |  |  | 2.13 |  |  |  | 2.25 |  |  |  | 2.26 |  |  |
| *N*1 | Median | 8520 |  |  |  | 5481 |  |  |  | 5671 |  |  |  | 3105 |  |  |
|  | Mean | 9136 |  |  |  | 5614 |  |  |  | 6321 |  |  |  | 3939 |  |  |
|  | q.05 | 1701 |  |  |  | 1368 |  |  |  | 1261 |  |  |  | 557 |  |  |
|  | q.95 | 62659 |  |  |  | 24864 |  |  |  | 48203 |  |  |  | 67233 |  |  |
| Log10(*N*1) | HPD 95 lower | 3.06 |  |  |  | 3.00 |  |  |  | 2.90 |  |  |  | 2.50 |  |  |
|  | HPD 95 upper | 4.93 |  |  |  | 4.52 |  |  |  | 4.82 |  |  |  | 4.99 |  |  |
|  | HPD 50 lower | 3.58 |  |  |  | 3.48 |  |  |  | 3.42 |  |  |  | 3.04 |  |  |
|  | HPD 50 upper | 4.20 |  |  |  | 3.98 |  |  |  | 3.99 |  |  |  | 3.73 |  |  |
|  | Mode | 3.74 |  |  |  | 3.78 |  |  |  | 3.56 |  |  |  | 3.26 |  |  |
| *T* | Median | 3859 | 6431 | 10290 |  | 204.031 | 340 | 544.083 |  | 848.613 | 1414 | 2262.97 |  | 1141.21 | 1902 | 3043.2 |
|  | Mean | 3255 | 5425 | 8680 |  | 168.748 | 281 | 449.995 |  | 727.367 | 1212 | 1939.65 |  | 903.543 | 1506 | 2409.4 |
|  | q.05 | 150 | 249 | 399 |  | 1139.59 | 4.2 | 149.607 |  | 236378 | 15.7 | 125.313 |  | 378205 | 4.6 | 1368.0 |
|  | q.95 | 42708 | 71180 | 113888 |  | 71179.9 | 9818 | 2.8852 |  | 113888 | 54348 | 1260.57 |  | 300.891 | 236378 | 9.4 |
| Log10(*T*) | HPD 95 lower | 1.97 | 2.19 | 2.39 |  | 0.15 | 0.37 | 0.57 |  | 0.69 | 0.91 | 1.12 |  | 0.03 | 0.25 | 0.46 |
|  | HPD 95 upper | 5.00 | 5.23 | 5.43 |  | 4.13 | 4.35 | 4.55 |  | 4.88 | 5.11 | 5.31 |  | 5.61 | 5.83 | 6.04 |
|  | HPD 50 lower | 3.19 | 3.42 | 3.62 |  | 1.75 | 1.97 | 2.18 |  | 2.33 | 2.55 | 2.76 |  | 2.27 | 2.49 | 2.69 |
|  | HPD 50 upper | 4.09 | 4.31 | 4.51 |  | 3.12 | 3.34 | 3.55 |  | 3.79 | 4.01 | 4.21 |  | 4.24 | 4.46 | 4.67 |
|  | Mode | 3.57 | 3.80 | 4.00 |  | 3.08 | 3.30 | 3.51 |  | 2.89 | 3.11 | 3.32 |  | 3.29 | 3.51 | 3.72 |
| *R* | Median | -1.59 |  |  |  | -2.47 |  |  |  | -2.01 |  |  |  | -1.53 |  |  |
|  | Mean | -1.64 |  |  |  | -2.66 |  |  |  | -2.17 |  |  |  | -1.66 |  |  |
|  | q.05 | -2.46 |  |  |  | -4.51 |  |  |  | -3.83 |  |  |  | -3.51 |  |  |
|  | q.95 | -0.93 |  |  |  | -1.39 |  |  |  | -1.09 |  |  |  | -0.33 |  |  |
|  | Var | 0.30 |  |  |  | 0.96 |  |  |  | 0.70 |  |  |  | 0.99 |  |  |
|  | BF H1 | 54.51 |  |  |  | 3.21 |  |  |  | 8.87 |  |  |  | 11.50 |  |  |
|  | BF H2 | 0.00 |  |  |  | 0.00 |  |  |  | 0.00 |  |  |  | 0.01 |  |  |

Values of quantile 0.05 (q.05), quantile 0.95 (q.95), mean and median of the three posterior distribution *N*0 (current effective population size), *N*1 (old effective population size before the demographic event), *T* (time in years elapsed since demographic event, for three different generation time (3, 5 and 8 years before present), see main text for discussion.) and R: (log_10_(*N*_0_/*N*_1_)), obtain from analysis on MSVAR 1.3 (Stortz and Beaumont 2002). For R, Variance (Var) and Bayes factor for population decline and population increase hypothesis (respectively BFH1, and BFH2) are given as measures of accuracy of the analysis see main text for description and Chikhi et al (2010) and Girod et al (2011) for comparisons.

***Table S6: Bibliographic review of C. newtoni’s population size estimates.***

| Year | Population size estimates | | Sex-ratio | Minimum high | Maximum high | Author commentary about abundance | Reference |
| --- | --- | --- | --- | --- | --- | --- | --- |
|  | Min | Max |  |  |  |  |  |
| 1865 |  |  |  | 800 | 1400 | "abbondant" (abundant) | Pollen, 1865 |
| 1880 |  |  |  |  |  | "imminent extinction" | Lanz (1887) in Cheke (1987) |
| 1946 |  |  |  |  |  | "virtuellement éteinte" (virtually extinct) | Berlioz, 1946 |
| 1948 |  |  |  | 600 |  | "en voie d'extinction" (in way to extinction) | Milon, 1951 |
| 1965 | 10 |  |  |  |  | "unlikely that more than about 10 pairs survive" | Vincent (1966) in Cheke (1987) |
| 1974-1975 | 120 | 150 |  | 1300 | 1800 |  | Cheke, 1976 |
| 1986 | 26 | 34 |  | 1400 | 1900 |  | Chazel, 1988 |
| 1987 | 24 | 60 |  | 1100 |  | "au bord de l'extinction" (nearly extinct) | Cherel, 1988 |
| 1999 | 120 | 150 |  | 1150 | 1850 |  | Probst, 1999 |
| 2003 | 100 | 100 | 1.37 | 1000 | 1800 |  | Ghestemme et al, 2007 |
| 2004 | 40 |  | 1.91 |  |  | Endangered (UICN, 2004) | SEOR, 2008 |
| 2005 | 43 |  | 2.36 |  |  |  | SEOR, 2008 |
| 2006 | 44 |  | 3.00 |  |  |  | SEOR, 2008 |
| 2007 | 47 |  | 2.22 |  |  |  | SEOR, 2008 |
| 2008 | 48 |  | 2.00 | 1300 | 1700 | Critically endangered (UICN, 2008) | SEOR, 2008 |

Population sizes are expressed in occupied territory units, sex ratio in number of male per female.

Berlioz J (1946). Faune de l'empire français, IV: oiseaux de La Réunion. Librairie Larose.

Chazel L (1988). Note sur le Tuit‐tuit, oiseau endémique de La Réunion. Rap. phot. pour ICBP, 10p.

Cheke AS (1976). Rapport sur la distribution et la conservation du Tuit-tuit, oiseau rarissime de La Réunion. Rapport

Cherel JF (1988) L'Echenilleur de La Réunion. Coracina newtoni, va-t-il disparaître? *Alauda* 56, 182.

Jouanin C (1973) Note sur l'avifaune de La Réunion. L'oiseau, *Revue Française d'Ornithologie* **34**, 83-84.

Milon Ph (1951) Notes sur l'avifaune actuelle de l'île de La Réunion. Terre et Vie **98 :** 128-178.

British Ornithologists Union Mascarene Island Expedition, *Conservation Memorendum* 2: 16 pp.

Pollen FPL (1865) Note sur l'Oxynotus ferrugineus. *Bulletin de la Société Acclimatation Histoire Naturelle de l'île de La Réunion*, St Denis.

Probst JM (1999) Recherche sur la distribution ancienne plausible de l’Echenilleur de la Réunion Coracina newtoni (Pollen, 1866). *Bulletin Phaethon*, *9* : 24-44.

SEOR (2008), Co-gestion de la Réserve de la Roche Ecrite, Bilan des activités, p. 31.

***Table S7: Extrapolation of ancient population size using vegetation type data from Strasberg et al. (2005).***

|  | Habitat Area (ha) | | Estimated Population Size | | |
| --- | --- | --- | --- | --- | --- |
|  | Current | Ancient | Current | Ancient Min | Ancient Max |
| H0 : actual repartition | 1250 | - | ~ 70 | 70 | 210 |
| H1 : same vegetation type | 12400 | 43366 |  | 2428 | 7286 |
| H2 : all upland wet forest | 61314 | 106925 |  | 5988 | 17965 |
| H3 : all wet forest | 66921 | 156190 |  | 8747 | 26243 |
| H4: all forest | 67466 | 224011 |  | 12545 | 37638 |

Ancient population size extrapolation are estimated by multiplying current population size by the ratio of ancient habitat over current habitat size (Ancient Min), for each selected vegetation type from Strasberg et al. (2005; [50]), Ancient Max values are obtained by multiplying Ancient Min values by a 3/2 factor to equilibrate ancient sex ratio (compare to current one) and by 2/1 factor to reduce home range size of a pair of individuals, as suggested by greater resources availability at lower altitudes and by home range size of other Coracina species [27,29].

***Table S8: C.newtoni sampling effort per year.***

| Sampling year | Nb of samples |
| --- | --- |
| 2003 | 4 |
| 2004 | 3 |
| 2006 | 8 |
| 2007 | 18 |
| 2008 | 13 |

**Table S9**: Starting values for MSVAR 0.4

|  | Starting values for | | |  | Lower and upper uniform prior bounds | | |  | Run lengths | | |
| --- | --- | --- | --- | --- | --- | --- | --- | --- | --- | --- | --- |
|  | log(*r*) | log*Ө* | log(*Tf*) |  | log(*r*) | log*Ө* | log(*Tf*) |  | Steps | Thinning | Iterations |
| Run 1 | 100 | 1 | 1 |  | -5, 5 | -5, 5 | -5, 5 |  | 1.E+05 | 5.E+04 | 5.E+09 |
| Run 2 | 100 | 1 | 1 |  | -5, 5 | -5, 5 | -5, 5 |  | 1.E+05 | 5.E+04 | 5.E+09 |
| Run 3 | 1000 | 10 | 0.1 |  | -5, 5 | -5, 5 | -5, 5 |  | 1.E+05 | 5.E+04 | 5.E+09 |
| Run 4 | 1000 | 10 | 0.1 |  | -5, 5 | -5, 5 | -5, 5 |  | 1.E+05 | 5.E+04 | 5.E+09 |
| Run 5 | 10000 | 0.1 | 1000 |  | -5, 5 | -5, 5 | -5, 5 |  | 1.E+05 | 5.E+04 | 5.E+09 |
| Run 6 | 10000 | 0.1 | 1000 |  | -5, 5 | -5, 5 | -5, 5 |  | 1.E+05 | 5.E+04 | 5.E+09 |

**Table S10: Starting values for MSVAR 1.3**

|  | Starting values (mean, variance) for | | | |  | Hyperpriors (α, σ, β, τ) for | | | |  | Run lengths | | |
| --- | --- | --- | --- | --- | --- | --- | --- | --- | --- | --- | --- | --- | --- |
|  | log(*N*_0_) | log(*N*_1_) | log*Ө* | log(*Tf*) |  | log(*N*_0_) | log(*N*_1_) | log*Ө* | log(*Tf*) |  | Steps | Thinning | Iterations |
| Run 1 | 4, 1 | 4, 1 | -3.5, 1 | 2, 1 |  | 3, 2, 0, 0.5 | 5, 3, 0, 0.5 | -3.5, 0.25, 0, 2 | 5, 3, 0, 0.5 |  | 5.E+05 | 5.E+04 | 3.E+10 |
| Run 2 | 4, 5 | 4, 5 | -3.5, 0.5 | 5, 5 |  | 3, 2, 0, 0.5 | 3, 3, 0, 0.5 | -3.5, 0.25, 0, 2 | 3, 3, 0, 0.5 |  | 5.E+05 | 5.E+04 | 3.E+10 |
| Run 3 | 4, 2 | 4,2 | -3.5, 1 | 5, 1 |  | 3, 2, 0, 0.6 | 5, 2, 0, 0.5 | -3.5, 0.25, 0, 0.5 | 5, 3, 0, 0.5 |  | 5.E+05 | 5.E+04 | 3.E+10 |
| Run 4 | 4, 1 | 4, 1 | -3.5, 1 | 5, 4 |  | 3, 3, 0, 0.5 | 5, 2, 0, 0.5 | -3.5, 0.25, 0, 2 | 5, 3, 0, 0.5 |  | 5.E+05 | 5.E+04 | 3.E+10 |

**Figure S1: Most likely population sub-structure.**

Results obtained with TESS (a) and confirmation from STRUCTURE (b) while using TESS individual’s attribution to clusters as prior information (see text for details). Samples are ranked from West to East.


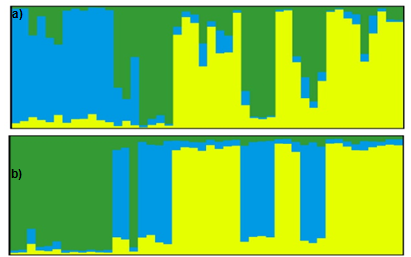


**Figure S2: Posterior distributions for the past (*N*1) and current (*N*0) effective population sizes, and time since the population collapse in years (T),** represented in log10 scale, for the three identified clusters. The solid lines correspond to the posterior distribution obtained by pooling independent MCMC run. The different priors used are shown for comparison for N0 and T, (dashed lines) and for N1 (doted- lines).

**
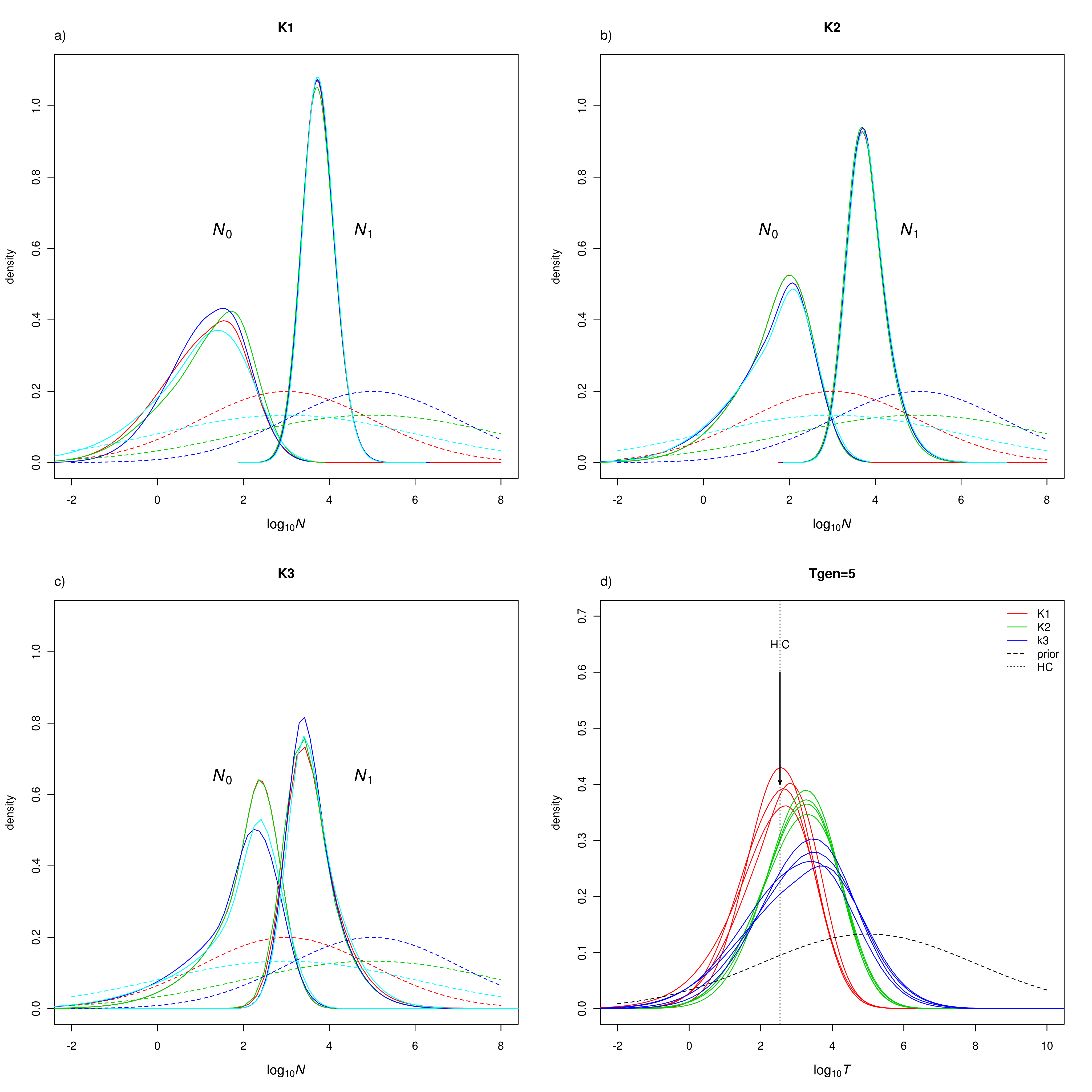
**
